# Tailoring pullulanase PulAR from *Anoxybacillus* sp. AR-29 for enhanced catalytic performance by a structure-guided consensus approach

**DOI:** 10.1186/s40643-022-00516-4

**Published:** 2022-03-21

**Authors:** Shu-Fang Li, Shen-Yuan Xu, Ya-Jun Wang, Yu-Guo Zheng

**Affiliations:** 1 Key Laboratory of Bioorganic Synthesis of Zhejiang Province, College of Biotechnology and Bioengineering, Zhejiang University of Technology, 18 Chaowang Road, Hangzhou, 310014 People’s Republic of China; 2 Engineering Research Center of Bioconversion and Biopurification of the Ministry of Education, Zhejiang University of Technology, Hangzhou, Zhejiang 310014 People’s Republic of China; 3 The National and Local Joint Engineering Research Center for Biomanufacturing of Chiral Chemicals, Zhejiang University of Technology, Hangzhou, 310014 People’s Republic of China

**Keywords:** Pullulanase, Structure-guided consensus approach, Site-directed mutagenesis, Catalytic pocket, Stability, Catalytic efficiency

## Abstract

Pullulanase is a well-known debranching enzyme that can specifically hydrolyze α-1,6-glycosidic linkages in starch and oligosaccharides, however, it suffers from low stability and catalytic efficiency under industrial conditions. In the present study, four residues (A365, V401, H499, and T504) lining the catalytic pocket of *Anoxybacillus* sp. AR-29 pullulanase (PulAR) were selected for site-directed mutagenesis (SDM) by using a structure-guided consensus approach. Five beneficial mutants (PulAR-A365V, PulAR-V401C, PulAR-A365/V401C, PulAR-A365V/V401C/T504V, and PulAR-A365V/V401C/T504V/H499A) were created, which showed enhanced thermostability, pH stability, and catalytic efficiency. Among them, the quadruple mutant PulAR-A365V/V401C/T504V/H499A displayed 6.6- and 9.6-fold higher catalytic efficiency toward pullulan at 60 ℃, pH 6.0 and 5.0, respectively. In addition, its thermostabilities at 60 ℃ and 65 ℃ were improved by 2.6- and 3.1-fold, respectively, compared to those of the wild-type (WT). Meanwhile, its pH stabilities at pH 4.5 and 5.0 were 1.6- and 1.8-fold higher than those of WT, respectively. In summary, the catalytic performance of PulAR was significantly enhanced by a structure-guided consensus approach. The resultant quadruple mutant PulAR-A365V/V401C/T504V/H499A demonstrated potential applications in the starch industry.

**Graphical Abstract:**

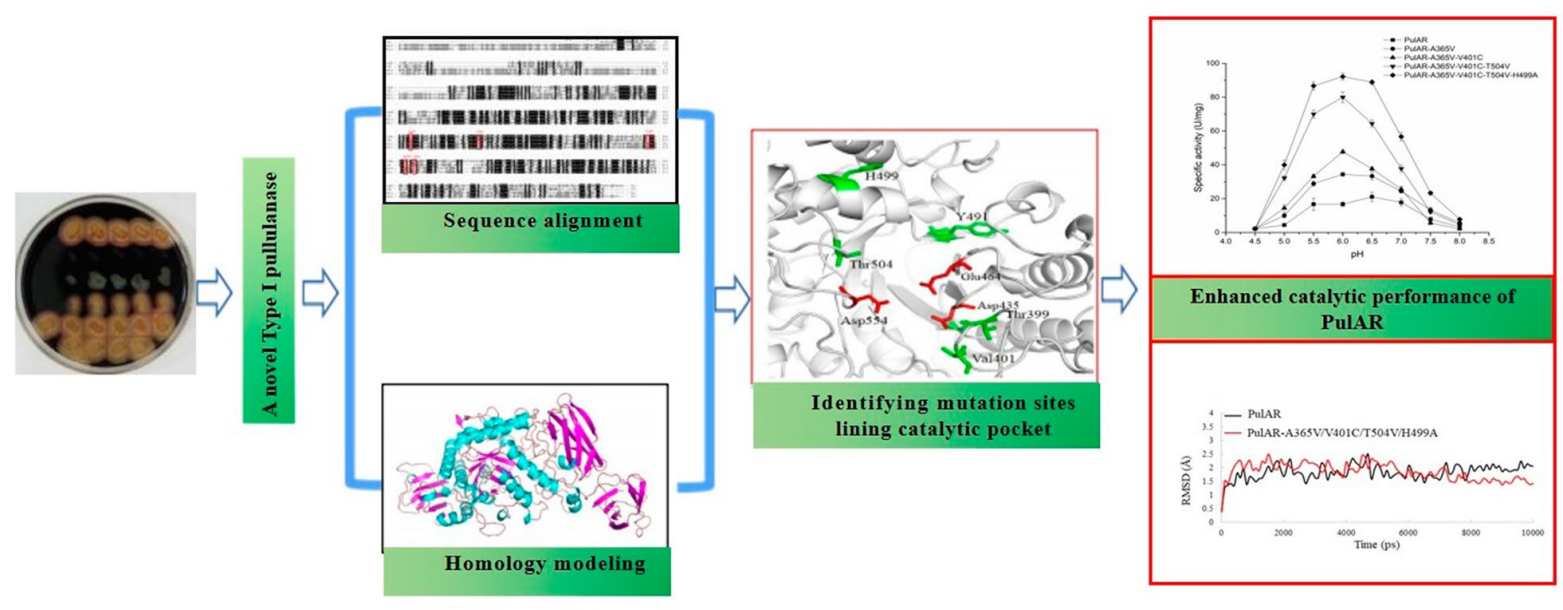

**Supplementary Information:**

The online version contains supplementary material available at 10.1186/s40643-022-00516-4.

## Introduction

Starch transformation can be accomplished by using an enzymatic process that involves two primary steps: liquefaction and saccharification (Hii et al. 2012). Generally, the saccharification is conducted at 60 ℃ and pH 4.5–5.5 for 48–60 h, via pullulanase in combination with β-amylase or glucoamylase, producing maltose syrup and glucose syrup. Pullulanase [EC 3.2.1.41] is a debranching enzyme that can specifically cleave α-1,6-glycosidic linkages in pullulan, starch, amylopectin, glycogen, and related oligosaccharides (Bertoldo and Antranikian 2002). Addition of pullulanase would reduce the amount of glucoamylase or β-amylase used in the saccharification step and improve substrate concentration and conversion (Duan et al. 2013). High-purity maltose syrup is a low-calorie, low-sweetness sugar that is being widely used in the food, medicine, and cosmetic industries (Bertoldo et al. 1999), in which the content of maltose is above 60%. In recent years, enzymatic preparation of maltose syrups has drawn rising interest for its mild reaction condition, high selectivity, and high catalytic efficiency (Lin et al. 2013). In combination with β-amylase and/or maltase, pullulanase can raise starch hydrolysis efficiency to high-purity maltose syrup and reduce production cost.

Pullulanases are divided into type I pullulanase and type II pullulanase based on substrate specificity and reaction products. Type II pullulanase hydrolyzes both α-1,6-glycosidic linkages and α-1,4-glycosidic linkages (Kang et al. 2011; Li et al. 2012; Pang et al. 2019). Compared with type II pullulanase, type I pullulanase specifically hydrolyzes α-1,6-glycosidic linkages in pullulan and other polysaccharides, forming maltotriose and linear oligomers. In recent years, a few type I pullulanases from *Fervidobacterium nodosum* Rt17-B1(Yang et al. 2020), *Bacillus methanolicus* PB1 (Zhang et al. 2020), *Geobacillus thermocatenulatus* DSMZ73010 (Li et al. 2018), *Bacillus megaterium* W1210 (Yang et al. 2017), *Anoxybacillus* sp. SK3-4 (Kahar et al. 2016) and *Paenibacillus polymyxa* Nws-pp2 (Wei et al. 2015) have been cloned and characterized. However, most of the reported type I pullulanases exhibit neutral or basic pH optimum, and their stabilities under acidic or thermophilic conditions are usually poor.

Protein engineering is an efficient way to obtain the desirable enzymes (Böttcher and Bornscheuer 2010). As reported previously, many reports focused on enhancing the thermostability or catalytic efficiency of the type I pullulanases. For example, Duan et al*.* successfully improved the thermostability and catalytic efficiency of a Type I pullulanase from *Bacillus deramificans* by site-directed mutagenesis (SDM) (Duan et al. 2013). In a recent example, Bi and coworkers employed a computer-aided method to raise *Tm* of the thermophilic pullulanase from *Bacillus thermoleovorans* by 3.8 ℃ (Bi et al. 2020). Up to date, only a limited number of reports on improving acidic adaptation of pullulanase are available (Wang et al. 2017; Zeng et al. 2019). Chen and coworkers improved the acidic adaptation of *Bacillus acidopullulyticus* pullulanase by altering hydrogen bonds network near the catalytic residues, shifting its optimum pH from 5.0 to 4.0 at the expense of activity reduction (Chen et al. 2019). Therefore, it is still needed to dig out the pullulanase with high catalytic efficiency and stability under thermophilic and acidic conditions.

In this study, we identified a novel pullulanase from *Anoxybacillus* sp. AR-29 (PulAR). Four residues A365, V401, H499, and T504 lining the catalytic pocket were identified as critical for the thermostability and acid resistance by a structure-guided consensus approach. The catalytic performance of PulAR under thermophilic and acidic conditions was enhanced by SDM. In addition, structural analysis and MD simulations were performed to elucidate their roles.

## Materials and methods

### Bacterial strains, plasmids, and enzyme

The *Anoxybacillus* sp. AR-29 strain was isolated and stored in our laboratory. The PulAR gene (GenBank accession number KY273924.1) was cloned from *Anoxybacillus* sp. AR-29. We have constructed the pET-32a ( +)-*PulAR* plasmid in our previous study. *Escherichia coli* DH5α was used as the host for the cloning work, and *E. coli* BL21(DE3) was the host for the expression of the enzymes. Phanta Super-Fidelity DNA Polymerase and the restriction enzyme *Dpn* I were purchased from Vazyme Biotech Co., Ltd (Nanjing, China). All other chemicals and reagents were obtained from standard commercial sources.

### Genomic DNA extraction, amplification and bioinformatics analysis

The genomic DNA of *Anoxybacillus* sp. AR-29 was extracted using TIANamp Bacteria DNA Kit (Tiangen, Beijing, China). And the genomic DNA of *Anoxybacillus* sp. AR-29 was used as the template for the amplification of the pulAR-encoding gene, using the forward primer 5ʹ-GCGATATCATGTATGAGGTCTTTTCC-3 ʹ and reverse primer 5 ʹ -GCCTCGAGTTATATGTGATTTGCTTTTT-3 ʹ, respectively. *PulAR* gene was amplified by PCR according to the following protocol: denaturation at 95 °C for 60 s, 20 cycles of (95 °C, 30 s; 55 °C, 30 s; 72 °C, 90 s), and a final extension at 72 °C for 10 min. The protein sequence and nucleotide sequence of PulAR were analyzed by using BLASTp and BLASTn (http://www.ncbi.nlm.nih.gov/), respectively. The MW and pI of this enzyme were predicted via the web server (http://web.expasy.org/compute_pi/).

### Cloning, over-expression and purification of PulAR

The PCR products were double digested with the restriction enzymes *EcoR* V and *Xho* I and cloned into the pET-32a ( +), which was also digested by the same restriction enzymes, yielding the recombinant plasmid pET-32a ( +)-*PulAR.* For over-expression of PulAR in *E. coli* BL21(DE3), the recombinant plasmid was transformed into *E. coli* BL21(DE3). The transformant was picked into the tube with 5 mL LB medium containing 100 μg/mL ampicillin and cultivated at 37 °C overnight. The overnight cultures were then transferred into another 100 mL of LB medium containing ampicillin (100 μg/mL), and cultivated for 3 h until the OD at 600 nm was between 0.6 and 0.8. The protein expression was then induced by adding IPTG at a final concentration of 0.5 mM for 16 h at 16 °C. The cells were harvested by centrifugation at 8000*g* for 10 min at 4 °C and resuspended in 20 mL of binding buffer (20 mM Tris–HCl, 250 mM NaCl, 20 mM imidazole). Cell lysates were prepared with a French press operating at 4 °C, and then centrifuged at 8000*g* for 30 min. The resultant soluble fraction was micro-filtrated, and loaded onto a Ni–NTA column which was pre-equilibrated with the binding buffer. The target protein was eluted by a 20–250 mM imidazole gradient at a flow rate of 1 mL/min. The protein was pooled and dialysed with Buffer C (20 mM Tris–HCl and 150 mM NaCl, pH 8.0). The purified protein was estimated by SDS-PAGE, and the concentration of the protein was determined by the BCA protein assay kit.

### Characterization of WT-PulAR

Pullulanase activity was measured in 500 μL reaction mixtures that contained 50 μL of pullulan (0.5%), 400 μL of sodium phosphate buffer (100 mM, pH 6.0), and the appropriate amounts of the purified enzymes, and incubated at 60 °C for 10 min. One unit of pullulanase activity was defined as the amount of enzyme required to release 1 μmol of reducing sugars per minute. Effects of pH on the purified PulAR were determined in 100 mM buffer over pH 3.6–9, including sodium acetate buffer (pH 3.6–5.8), sodium phosphate buffer (pH 5.8–7.5) and Tris–HCl buffer (pH 7.5–9.0). The temperature optimum of PulAR was measured at temperatures between 45 and 95 °C in 100 mM sodium acetate buffer (pH 6.0).

The kinetic parameters of WT-PulAR were determined according to the method as previously described (Li et al. 2015), using pullulan at varying concentrations (1.0, 1.25, 1.33, 2.0, 2.5, 3.33, and 5.0 mg/mL) as substrate at 60 °C for 10 min in 100 mM buffer (pH 6.0). Experiments were conducted in triplicates. The Michaelis–Menten equation was fitted to the data points to determine *K*_M_ and *v*_max_ by nonlinear least-squares regression analysis using Origin 8.5.

### Screening for hotspot residues by a structure-guided consensus approach

The protein sequence of PulAR was aligned with the pullulanases from *Anoxybacillus* sp. 18–11 (pH_opt_ 6.0) (PulA) (Xu et al. 2014), *Bacillus acidopullulyticus* (pH_opt_ 5.0) (*Ba*pul) (Turkenburg et al. 2009), and *Bacillus naganoensis* (pH_opt_ 4.5) (*Bn*pul) (Nie et al. 2013). The temperature optimum range of these pullulanases was 55–65 °C. The putative structure of PulAR was obtained with the homology-modeling pipeline SWISS-MODEL server (http://swiss-model.expasy.org), using the structure of type I pullulanase (PDB ID: 3WDH) from *Anoxybacillus* sp. LM18-11 as the template. The structures were analyzed and visualized by using PyMOL (http://www.pymol.org/). The residues within 8 Å of the catalytic triad of PulAR were identified and the differences in these amino acid residues among the above pullulanases were explored. Totally, five residues (A365, T399, V401, Y491, and T504) different from those of the acidophilic pullulanases (*Ba*pul and *Bn*pul) were selected for SDM. In addition, the mutation Y477A could improve the thermostability of a Type I pullulanase PulA in our previous report (Li et al. 2015). Therefore, the residue H499 of PulAR was also chosen for SDM, which was corresponding to Y477 of PulA.

### Construction of the mutants

The PulAR gene was cloned into the pET-32a( +) plasmid, and the recombinant plasmid pET-32a( +)-*PulAR* was used as the template for site-directed mutagenesis. The PCR was conducted as follows: 95 °C for 5 min, then 26 cycles (95 °C for 30 s, 50 °C for 30 s, and 72 °C for 8 min), and final extension at 72 °C for 10 min. The PCR reaction system (25 μL) consisted of 12.5 μL 2 × Phanta buffer, 0.5 μL dNTP mixture (each at 10 mmol L^−1^), 1 μL forward primer (10 μmol L^−1^), 1 μL reverse primer (10 μmol L^−1^), 1 μL plasmid template (50 ng), 8.5 μL ultra-pure water, and 0.5 μL DNA polymerase (1 U μL^−1^). The primers are listed in Additional file 1: Table S1. The PCR products were digested with *Dpn* I and then transformed into *E. coli* BL21(DE3). To verify that only the designated mutations were inserted by the DNA polymerase, the full plasmids containing the pullulanase gene were sequenced.

### Expression, purification and characterization of PulAR mutants

Expression, purification, and characterization of the PulAR mutants were conducted according to the methods described above as 2.3 and 2.4. The purified protein was analyzed by SDS-PAGE (Additional file 1: Fig. S1).

### Structural and MD simulation analyses of PulAR

Homology modeling of the PulAR mutants was performed with the same approach as used for WT. Discovery Studio and DSSP web server (http://www.cmbi.ru.nl/xssp/) were adopted to analyze the structural information. YASARA software was used for molecular dynamics simulation (MD simulation). MD simulations were performed at 60 °C and pH 5.0 for 20 ns. During the dynamic simulation, the force field was Amber 03, the TIP3P model was used, and the concentration of NaCl was set at 0.9%. After initial minimization through the steepest descent and simulated annealing, convergence was reached. The time step is 1 fs, and the track is saved every 100 ps. All independent MD simulations were repeated three times.

## Results and discussion

### Sequence analysis of PulAR encoding gene

**Fig. 1:**
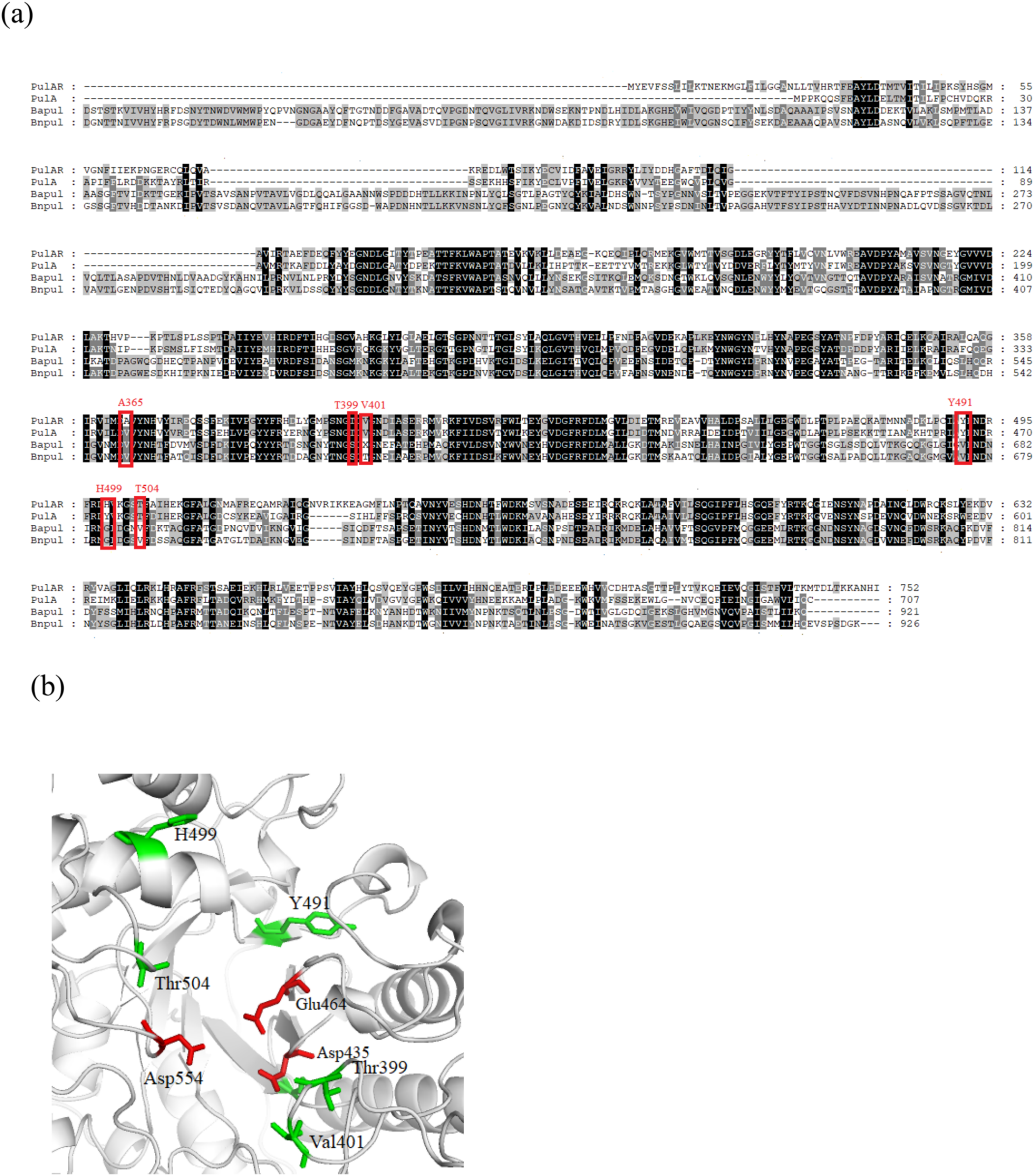
**a** Sequence alignment of PulAR with *Bacillus acidopullulyticus* pullulanase (*Ba*pul) and *Bacillus naganoensis* pullulanase (*Bn*pul). **b** Mutation hotspots A365, T399, V401, Y491, and T504

The pullulanase PulAR gene (2,259 bp long, GenBank accession number KY273924.1) has a putative translational start site GTG with a G + C content of 78.4%, and encodes an enzyme with a predicted molecular mass of 85.0 kDa with a theoretical pI of 5.49. The structure of PulAR was constructed based on the crystal structure of the pullulanase PulA from *Anoxybacillus* sp. LM18-11 (PDB ID: 3WDH), with which it shares 58.29% identity (Fig. 1A). Analysis of the protein sequence of PulAR by NCBI BLASTp showed that it contains the YNWGYDP motif and four conserved regions (I–IV) (Additional file 1: Fig. S2), which are similar to those of type I pullulanases and comprise a catalytic triad and several substrate binding sites. Therefore, the residues D435, E464, and D554 of PulAR are inferred as the catalytic residues. No signal peptide was found in the pullulanase PulAR through analysis by Signal P (https://services.healthtech.dtu.dk/service.php?SignalP-4.1). Comparison with the pullulanase sequences in the GenBank database, listed in Table 1, revealed that PulAR shares 70.8%, 60.2%, 58.3%, 46.5%, 43.6%, 41.5%, 41.1% and 38.1% identity with the thermostable pullulanases from *Bacillus stearothermophilu*s (Kuriki et al. 1990), *Geobacillus thermoleovorans* (Zouari Ayadi et al. 2008), *Anoxybacillus* sp. LM18-11 (Xu et al. 2014), *Bacillus* sp. CICIM 263 (Li et al. 2012), *Anaerobranca gottschalkii* (Bertoldo et al. 2004), *Fervidobacterium pennivorans* DSM 9078 (Bertoldo et al. 1999), *Thermotoga neapolitana* (Kang et al. 2011), and *Caldicellulosiruptor saccharolyticus* (Albertson et al. 1997), respectively.

**Table 1:** Comparison of optimum temperatures between bacterial type I pullulanases

Bacterial source	Accession number	Optimum temperature (℃)	Similarity with PulAR	Special activity	References
*Bacillus stearothermophilus*	1808262A	60	70.8%	0.214 U/mL	Kuriki et al. (1990)
*Geobacillus thermoleovorans*	CAC85704.1	70	60.2%	36 U/mg	Zouari Ayadi et al. (2008)
*Anoxybacillus* sp. LM18-11	AEW23439.1	60	58.3%	*v*_max_ 750 U/mg	Xu et al. (2014)
*Bacillus* sp. CICIM 263	AGA03915.1	70	46.5%	73 U/mg	Li et al. (2012)
*Anaerobranca gottschalkii*	AAS47565.1	65–70	43.6%	56 U/mg	Bertoldo et al. (2004)
*Fervidobacterium pennivorans* DSM 9078	AAD30387.1	80	41.5%	78 U/mg	Bertoldo et al. (1999)
*Thermotoga neapolitana*	ACN58254.1	80–85	41.1%	25.1 U/mg	Kang et al. (2011)
*Caldicellulosiruptor saccharolyticus*	AAB06264.1	85	38.1%	–	Albertson et al. (1997)
*Anoxybacillus* sp. AR-2	KY273924	55	100.0%	24.4 U/mg	This study
	KY273924	65	99.0%	87.8U/mg (PulAR-A365V/V401C/T504V/H499A)	This study

### Screening for mutation hotspots

To identify the critical residues responsible for catalytic activity and stability of PulAR, we compared the protein sequences of neutrophilic type I pullulanases (PulA and PulAR) with the acidophilic pullulanases (*Bn*Pul and *Ba*Pul) (Fig. 1a), which have temperature and pH optima of 50–60 °C and pH 4.5–6.0. The differences in the amino acid residues among the above pullulanases within 8 Å of the catalytic triad were explored (Fig. 1b), and six mutants A365V, T399S, V401T, V401C, Y491V, and T504V, were generated. Besides, the single mutant H499A was also constructed.

### Generation of PulAR-positive mutants and enzymatic characterization

Firstly, the pullulanase activities of seven mutants were assayed at pH 5.0 and pH 6.0, respectively, and then the activity ratio of WT and its mutants at pH 5.0 to that at pH 6.0 (*A*_pH5.0_/*A*_pH6.0_) were evaluated. As described in Additional file 1: Table S2, *A*_pH5.0_/*A*_pH6.0_ of WT-PulAR and its mutants (A365V, T399S, V401T, V401C, Y491V, T504V and H499A) were 0.20, 0.49, 0.12, 0, 0.75, 0.19, 0.29 and 0.50, respectively. Therefore, we combined the positive mutations A365V, V401C, T504V, and H499A, generating three triple mutants PulAR-A365V-V401C, PulAR-A365V-V401C-T504V, and PulAR-A365V-V401C-T504V-H499A.

**Fig. 2:**
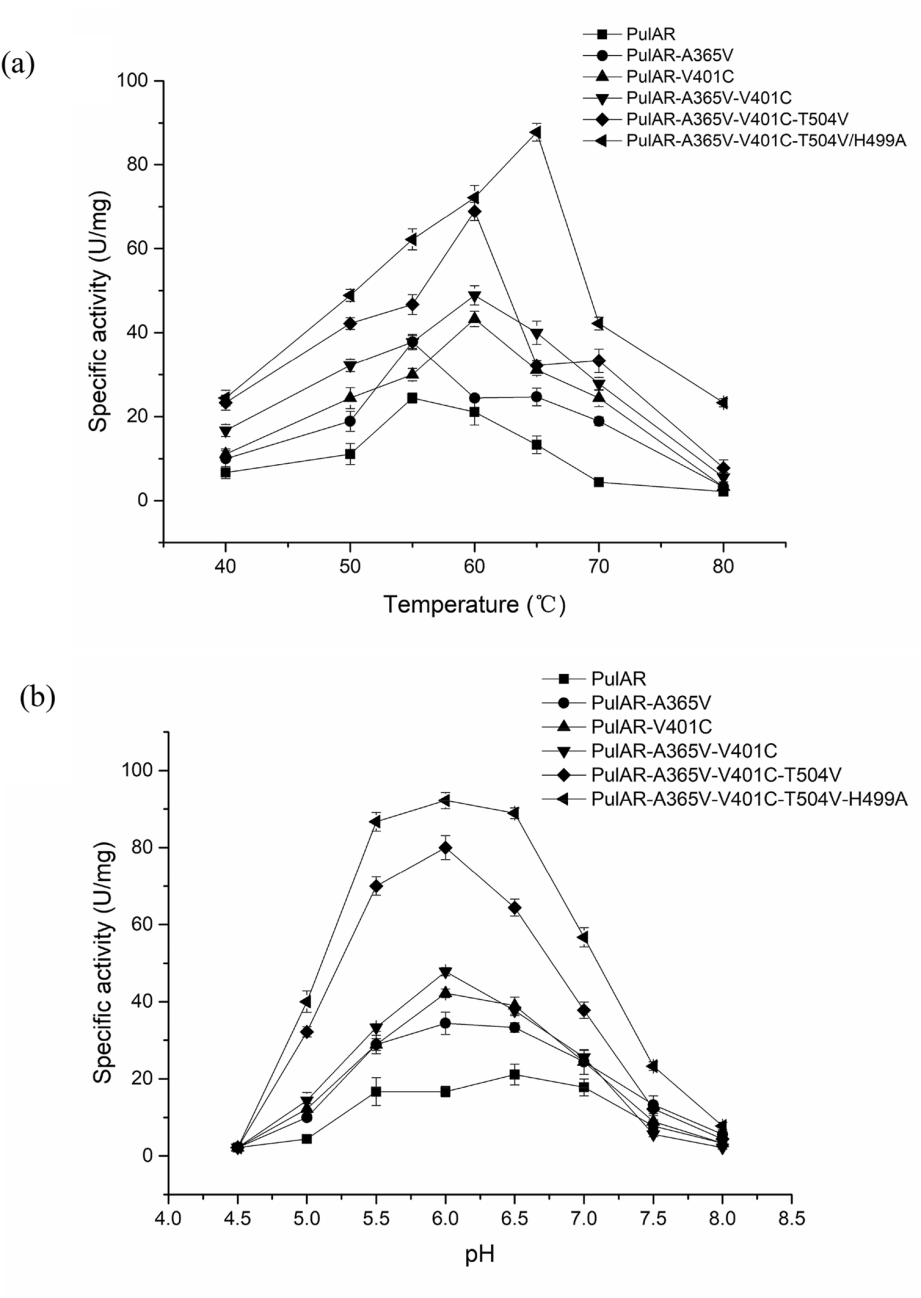
Temperature (**a**) and pH (**b**) optima of WT-PulAR and its mutants

We characterized the three combined mutants PulAR-A365V-V401C, PulAR-A365V-V401C-T504V, and PulAR-A365V-V401C-T504V-H499A, as well as two single mutants PulAR-A365V and PulAR-V401C. As shown in Fig. 2, the optimum temperature (*T*_opt_) of PulAR was 55 ℃, and these of the mutants PulAR-A365V, PulAR-V401C, PulAR-A365V-V401C, PulAR-A365V-V401C-T504V, and PulAR-A365V-V401C-T504V-H499A were 55, 60, 60, 60, and 65 ℃, respectively. Compared with WT, the *T*_opt_ of the mutant PulAR-A365V-V401C-T504V-H499A was increased by 10 ℃. In addition, at 60 ℃ and pH 6.0, the specific activities of WT and its mutants PulAR-A365V, PulAR-V401C, PulAR-A365V-V401C, PulAR-A365V-V401C-T504V, and PulAR-V401C-T504V-H499A were 24.4, 37.8, 43.3, 48.9, 68.9, and 87.8 U/mg. The optimum pH of PulAR-A365V, PulAR-V401C, PulAR-A365V-V401C, and PulAR-A365V-V401C-T504V was 6.0, which was similar to that of the WT. At 60 ℃ and pH 5.0, the specific activities of PulAR and its mutants PulAR-A365V, PulAR-V401C, PulAR-A365V-V401C, PulAR-A365V-V401C-T504V, and PulAR-A365V-V401C-T504V-H499A were 4.4, 10.0, 14.4, 32.2, and 40.0 U/mg, respectively. Among them, the specific activity of the quadruple mutant PulAR-A365V-V401C-T504V-H499A was 8.1-fold higher than that of WT at 60 ℃, pH 5.0.

**Fig. 3:**
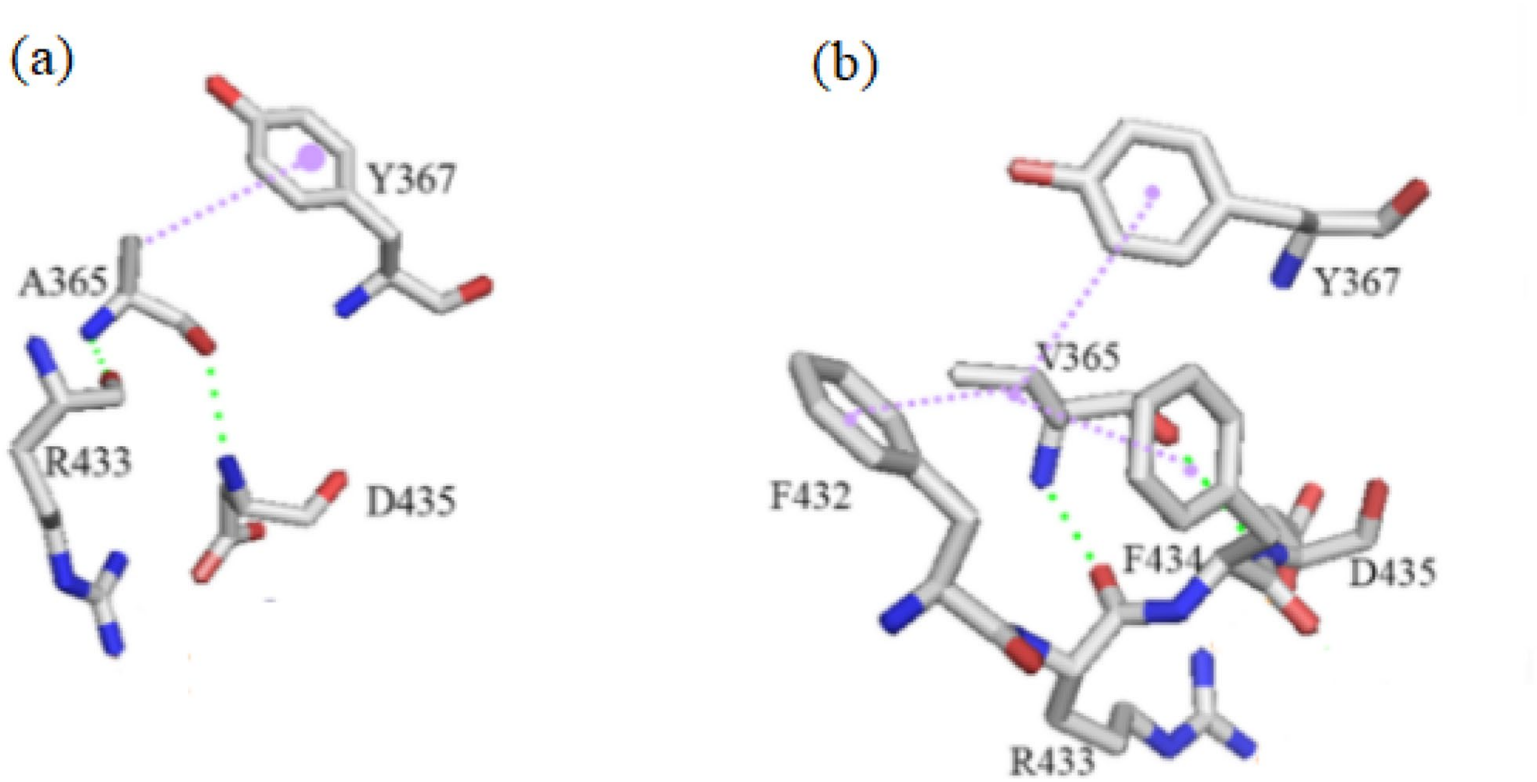
Structural analysis of PulAR before (**a**) and after (**b**) mutation A365V

**Fig. 4:**
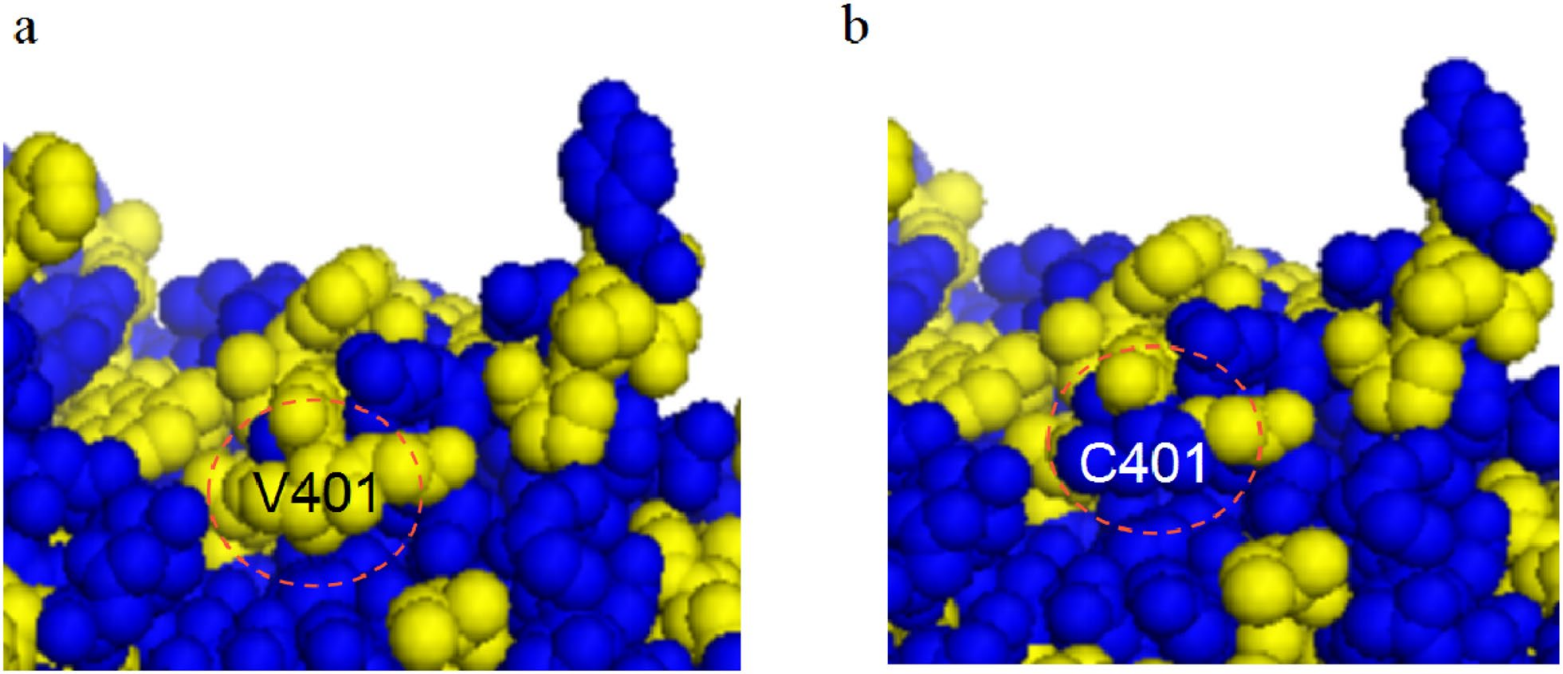
The hydrophobic residue V401 is shown in yellow (**a**) and the hydrophilic residue C401 is shown in blue (**b**)

To evaluate the thermostabilities of PulAR and its mutants, the enzymes were incubated at 60 ℃ and pH 6.0, and then the residual activities were assayed after varying incubation times. As shown in Table 2, all the mutants PulAR-A365V, PulAR-V401C, PulAR-A365V-V401C, PulAR-A365V-V401C-T504V, and PulAR-A365V-V401C-T504V-H499A displayed increased half-lives. At 60 and 65 °C, the half-lives (*t*_1/2_) of PulAR were only 4.8 and 2.5 h, respectively, whereas those of the quadruple mutant were 17.5 and 10.3 h, which were 2.65 and 3.12-fold higher than those of PulAR, respectively. The stabilities of PulAR under the acidic conditions (pH 4.5 and 5.0) were also significantly enhanced. The half-lives of PulAR were 5.4 and 6.1 h at pH 4.5 and 5.0, respectively, whereas those of the quadruple mutant PulAR-A365V-V401C-T504V-H499A displayed longer half-lives of 13.9 and 17.3 h, respectively (Table 3). The structures of PulAR and its mutants were modeled to investigate the mechanisms of the enhanced thermostability and pH stability. Ala365 is buried in the internal of the protein. As shown in Fig. 3, the mutation A365V introduces two extra hydrophobic interactions F432-V365 and F434-V365 while maintaining the two hydrogen bonds V365-R433 and D435-V365. The residue V401 is located on the protein surface. The mutation V401C increased the hydrophilicity of the protein surface (Fig. 4), contributing to enhanced thermostability (Yu et al. 2012). Similar to the mutation Y477A in our previous report, the solvent accessibility of the residue at the position 499 was reduced from 29.9 to 2.4 as assayed by the DSSP web server, which might enable the protein structure of PulAR more compact (Li et al. 2005, 2015). Replacing T504 with Val possessing an extra CH_3_ group reinforces the hydrophobic interior of the structure, leading to enhancement of the thermostability and pH stability (Tai et al. 2011).

**Table 2:** Half-lives of WT-PulAR and its variants at 6065 °C and 65 °C

Mutant	60 °C		65 °C	
	*k*_*d*_ (1/h)	*t*_1/2_ (h)	*k*_*d*_ (1/h)	*t*_1/2_ (h)
WT-PulAR	0.14	4.8 ± 0.2	0.28	2.5 ± 0.1
PulAR-A365V	0.12	5.9 ± 0.5	0.17	4.1 ± 0.2
PulAR-V401C	0.12	6.0 ± 0.1	0.17	4.1 ± 0.1
PulAR-A365V-V401C	0.07	9.9 ± 0.2	0.10	6.7 ± 0.4
PulAR-A365V-V401C-T504V	0.05	13.2 ± 0.1	0.08	9.2 ± 0.2
PulAR-A365V-V401C-T504V-H499A	0.04	17.5 ± 0.1	0.07	10.3 ± 0.3

**Table 3:** Half-lives of WT-PulAR and its variants at pH 4.5 and 5.0

Mutant	pH 4.5		pH5.0	
	k_d_ (1/h)	*t*_1/2_ (h)	*k*_d_ (1/h)	*t*_1/2_ (h)
WT-PulAR	0.13	5.4 ± 0.3	0.11	6.1 ± 0.5
PulAR-A365V	0.10	7.0 ± 0.4	0.08	8.5 ± 1.4
PulAR-V401C	0.10	7.1 ± 0.6	0.08	8.4 ± 0.3
PulAR-A365V-V401C	0.07	9.4 ± 0.3	0.06	10.8 ± 0.2
PulAR-A365V-V401C-T504V	0.06	11.1 ± 0.4	0.05	13.6 ± 1.0
PulAR-A365V-V401C-T504V-H499A	0.05	13.9 ± 1.2	0.04	17.3 ± 1.2

### Catalytic efficiency measurement and MD analysis

WT-PulAR and its mutants were subjected to kinetic analysis at 60 ℃, pH 5.0 and 6.0, respectively. Compared with WT, at pH 6.0, 60 °C, the *K*_M_ values of PulAR-A365V, PulAR-V401C, PulAR-A365V-V401C, PulAR-A365V-V401C-T504V, and PulAR-A365V-V401C-T504V-H499A decreased by 7.3%, 7.3%, 24.4%, 33.5%, and 53.0%, respectively, while the *k*_cat_ values increased by 39.1%, 54.3%, 168.9%, 193.7%, and 254.3%, respectively (Table 4). In addition, at pH 5.0, 60 °C, the *K*_M_ values of PulAR-A365V, PulAR-V401C, PulAR-A365V-V401C, PulAR-A365V-V401C-T504V, and PulAR-A365V-V401C-T504V-H499A decreased by 15.2%, 19.3%, 31.7%, 43.7%, and 68.5%, respectively, while the *k*_cat_ values increased by 37.7%, 45.7%, 154.8% 196.7%, and 230.7%, respectively (Table 5). Resultantly, the catalytic efficiencies (*k*_cat_/*K*_M_) of the “best” quadruple mutant PulAR-A365V-V/V-H499A were 6.6- and 9.6-fold higher than those of PulAR, at pH 6.0 and 5.0, respectively. The catalytic efficiency of PulAR was enhanced by mutations, which were identified by sequence alignment of the acidophilic pullulanase and neutrophilic pullulanase. Further, the roles of A365, V401, T504, and H499 in the structure–function relationship were analyzed. A365, V401, T504 and H499 form the catalytic pocket, shown in Fig. 1b. They are located within 8 Å of the catalytic residues D435, E464, and D554. And all the single mutation A365V, V401C, T504V, H499, and the superposition of mutations tends to confer increased flexibility of the active sites, resulting in the increased catalytic efficiencies (Tables 4, 5).

**Table 4:** Catalytic efficiencies of WT-PulAR and its mutants at 60 °C and pH 6.0

Mutant	*v*_max_ (μmol min^−1^ mg^−1^)	*K*_M_ (mg mL^−1^)	*k*_cat_ (s^−1^)	*k*_cat_/*K*_M_ (mL mg^−1^ s^−1^)
WT-PulAR	31.6 ± 1.2	1.64 ± 0.20	52.7	32.1
PulAR-A365V	44.0 ± 2.3	1.52 ± 0.12	73.3	48.2
PulAR- V401C	48.8 ± 1.6	1.52 ± 0.50	81.3	54.2
PulAR-A365V-V401C	85.0 ± 2.5	1.24 ± 0.32	141.7	114.3
PulAR-A365V-V401C-T504V	92.9 ± 2.0	1.09 ± 0.11	154.8	142.0
PulAR-A365V-V401C-T504V-H499A	112.0 ± 3.1	0.77 ± 0.15	186.7	242.5

**Table 5:** Catalytic efficiencies of WT-PulAR and its mutants at 60 °C and pH 5.0

Mutant	*v*_max_ (μmol min^−1^ mg^−1^)	*K*_M_ (mg mL^−1^)	*k*_cat_ (s^−1^)	*k*_cat_/*K*_M_ (mL mg^−1^ s^−1^)
WT-PulAR	25.6 ± 1.1	4.67 ± 0.12	42.7	9.1
PulAR-A365V	35.3 ± 0.3	3.96 ± 0.13	58.8	14.8
PulAR-V401C	37.3 ± 0.5	3.77 ± 0.20	62.2	16.8
PulAR-A365V-V401C	65.3 ± 1.2	3.19 ± 0.50	108.8	34.1
PulAR-A365V-V401C-T504V	76.0 ± 2.3	2.63 ± 0.28	126.7	48.2
PulAR-A365V-V401C-T504V-H499A	84.7 ± 1.6	1.47 ± 0.05	141.2	96.1

**Fig. 5:**
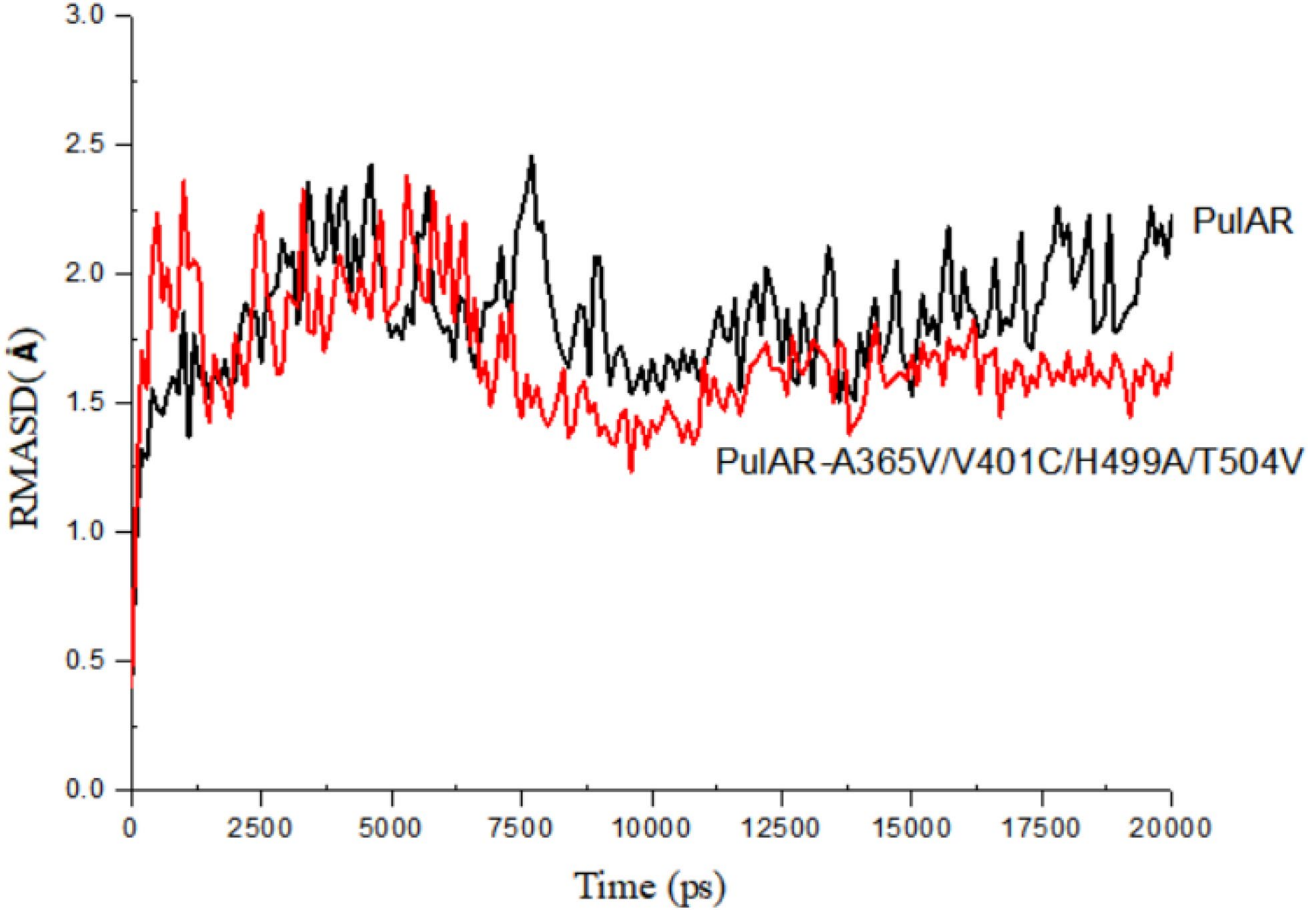
MD simulation analysis of WT-PulAR and its quadruple mutant PulAR-A365V-V401C-T504V-H499A

To further investigate the mechanism of the PulAR mutant against high temperature and acidic pH, MD simulation analysis of PulAR and the quadruple mutant PulAR-A365V-V401C-T504V-H499A was conducted. As shown in Fig. 5, during the initial 6 ns for simulation, both the structures of PulAR and quadruple mutant PulAR-A365V-V401C-T504V-H499A are unstable. The entire protein conformation of the quadruple mutant became more stable than PulAR after 6 ns simulation time, which was consistent with stability enhancement under the thermophilic and acidic conditions.

In this work, the catalytic performance of PulAR under the thermophilic and acidic conditions was significantly enhanced by using a structure-guided consensus approach. Four mutations A365V, V401C, T504V, and H499A were obtained by SDM. Finally, the “best” quadruple mutant PulAR-A365V/V401C/T504V/H499A showed higher catalytic activity and thermostabilities under the thermophilic and acidic conditions. Structural comparison indicated that the increased internal hydrophobic interactions, the reduced solvent accessibility surface area and the increased hydrophilic of the protein surface are the main reasons for the enhancement of thermostability and acid resistance. The “best” mutant PulAR-A365V/V401C/T504V/H499A exhibited great potential in the production of high-purity maltose syrup and other related starch processing industry.

### Supplementary Information

**Additional file 1:** **Table S1. **Primers design for site-directed mutagenesis. **Table S2. **Activity ratios of PulAR and its mutants at pH 5.0 to at pH 6.0. **Fig. S1.** SDS-PAGE analysis of WT-PulA and its mutants. M, Markers; Lane 1, purified WT-PulA; Lane 2, purified PulA-A365V; Lane 3, purified PulA-V401C; Lane 4, purified PulA-A365V/V401C; Lane 5, purified PulA-A365V/V401C/T504C; Lane 6, purified PulA-A365V/V401C/T504C/H499A. **Fig. S2.** Multiple sequence alignment of pullulanases from *Anoxybacillus* sp. AR-29, *Anoxybacillus* sp. LM18-11, *Bacillus acidopullulyticus *and *Bacillus naganoensis*. Conserved residues are indicated in frames.

## Data Availability

The data and the materials are all available in this article and additional document file.
