# Supplementary material for: Tailoring pullulanase PulAR from Anoxybacillus sp. AR-29 for enhanced catalytic performance by a structure-guided consensus approach
Source: Bioresour Bioprocess. 2022 Mar 21;9(1):25. doi: 10.1186/s40643-022-00516-4 (PMC10992289; doi:10.1186/s40643-022-00516-4)
Supplement: Supplementary file 1 — Additional file 1: Table S1. Primers design for site-directed mutagenesis. Table S2. Activity ratios of PulAR and its mutants at pH 5.0 to at pH 6.0. Fig. S1. SDS-PAGE analysis of WT-PulA and its mutants. M, Markers; Lane 1, purified WT-PulA; Lane 2, purified PulA-A365V; Lane 3, purified PulA-V401C; Lane 4, purified PulA-A365V/V401C; Lane 5, purified PulA-A365V/V401C/T504C; Lane 6, purified PulA-A365V/V401C/T504C/H499A. Fig. S2. Multiple sequence alignment of pullulanases from Anoxybacillus sp. AR-29, Anoxybacillus sp. LM18-11, Bacillus acidopullulyticus and Bacillus naganoensis. Conserved residues are indicated in frames. [file 40643_2022_516_MOESM1_ESM.docx]

**Additional information**

**Tailoring pullulanase PulAR from *Anoxybacillus* sp. AR-29 for enhanced catalytic performance by a structure-guided consensus approach**

Shu-Fang Li,^1, 2, 3^ Shen-Yuan Xu,^1, 2, 3^ Ya-Jun Wang,^1, 2, 3*^ Yu-Guo Zheng^1, 2, 3^

^1^ Key Laboratory of Bioorganic Synthesis of Zhejiang Province, College of Biotechnology and Bioengineering, Zhejiang University of Technology, Hangzhou 310014, P. R. China.

^2^ Engineering Research Center of Bioconversion and Biopurification of the Ministry of Education, Zhejiang University of Technology, Hangzhou, Zhejiang 310014, P. R. China.

^3^ The National and Local Joint Engineering Research Center for Biomanufacturing of Chiral Chemicals, Zhejiang University of Technology, Hangzhou 310014, P. R. China.

* Corresponding author:

Prof. Ya-Jun Wang

College of Biotechnology and Bioengineering

Zhejiang University of Technology

18 Chaowang Road, Hangzhou 310014, China

E-mail: wangyj@zjut.edu.cn, yajun246@hotmail.com

Tel: +86-571-88320957; Fax: +86-571-88320630

**Table S1** Primers design for site-directed mutagenesis

| Mutation | Primer sequences (5’-3’) | |
| --- | --- | --- |
| A365V | Forward primer | CGCGTGATTATGGATGTGGTTTATAATCAT |
|  | Reverse primer | CACATCCATAATCACGCGAATGCCCT |
| V401T | Forward primer | CCAATGGAACAGGAACTGGGAATGACA |
|  | Reverse primer | AGTTCCTGTTCCATTGGATGGCATTCCATAT |
| V401C | Forward primer | TCCAATGGAACAGGATGCGGGAATGACA |
|  | Reverse primer | GCATCCTGTTCCATTGGATGGCATTCCATAT |
| Y491V | Forward primer | CTGCCTTGTATTGCGGTGTTTAATGACA |
|  | Reverse primer | CACCGCAATACAAGGCAGCTTGTCAGCG |
| H499A | Forward primer | GACAGATTTCGCGATGCTGTCAAAGGAA |
|  | Reverse primer | AGCATCGCGAAATCTGTCATTAAAATACGCA |
| T504V | Forward primer | CATGTCAAAGGAAGTGTGTTTGCTATTC |
|  | Reverse primer | CACACTTCCTTTGACATGATCGCGAAAT |

**Table S2** Activity ratios of PulAR and its mutants at pH 5.0 to at pH 6.0

| Mutants | A_pH5.0_/A_pH6.0_^a^ |
| --- | --- |
| PulAR | 0.20 ± 0.012 |
| A365V | 0.49 ± 0.01 |
| T399S | 0.12± 0.006 |
| V401C | 0.75 ± 0.02 |
| V401T | 0 |
| Y491V | 0.19 ± 0.009 |
| T504V | 0.20 ± 0.012 |
| H499A | 0.50 ±0.002 |

^a^A_pH5.0_/A_pH6.0_, ratio of activity at pH 5.0 to activity at pH 6.0.


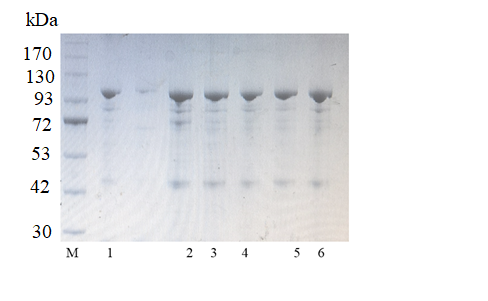


**Figure S1.** SDS-PAGE analysis of WT-PulA and its mutants. M, Markers; Lane 1, purified WT-PulA; Lane 2, purified PulA-A365V; Lane 3, purified PulA-V401C; Lane 4, purified PulA-A365V/V401C; Lane 5, purified PulA-A365V/V401C/T504C; Lane 6, purified PulA-A365V/V401C/T504C/H499A.


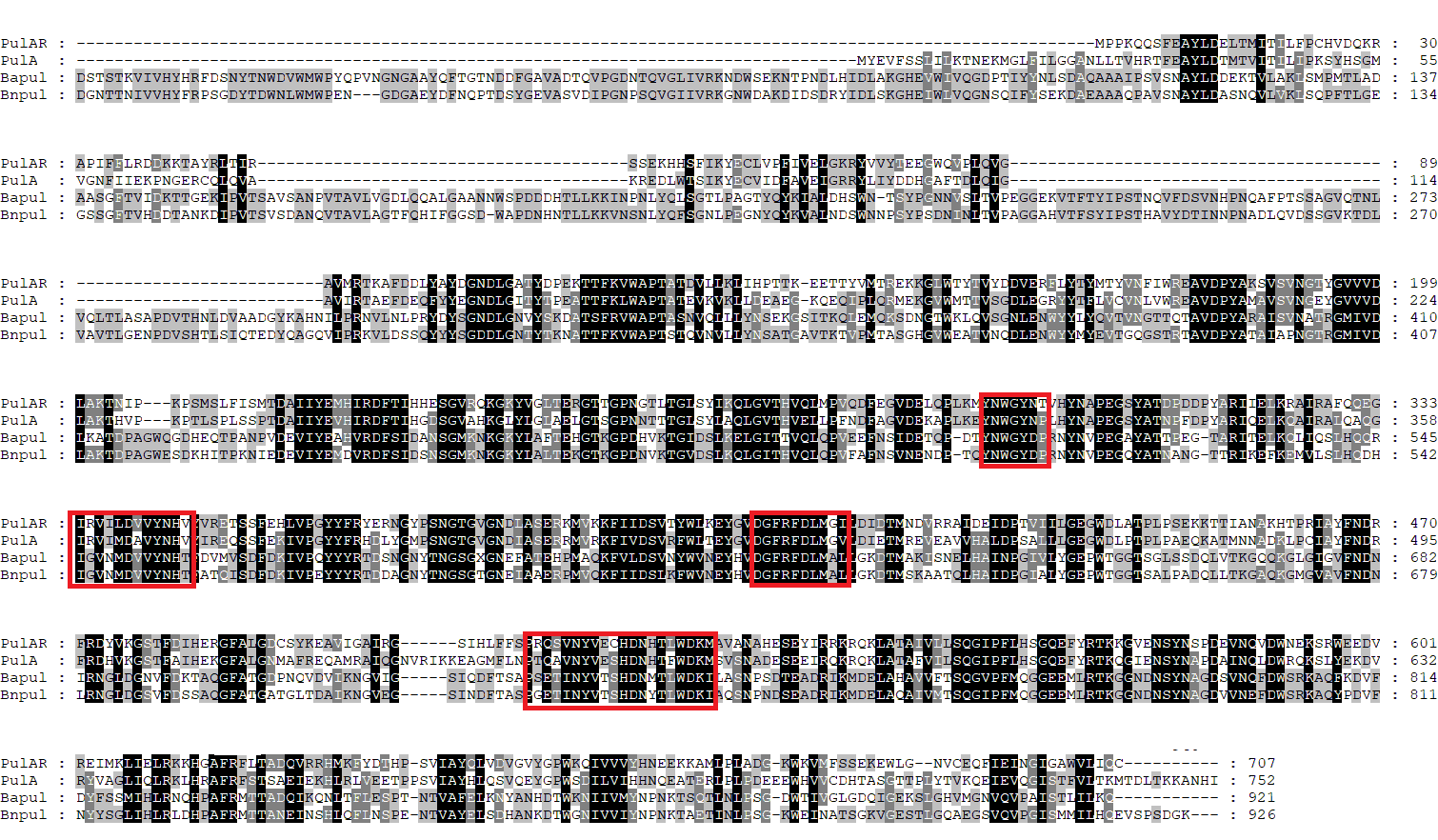


**Figure S2.** Multiple sequence alignment of pullulanases from *Anoxybacillus* sp. AR-29, *Anoxybacillus* sp. LM18-11*, Bacillus acidopullulyticus* and *Bacillus naganoensis*. Conserved residues are indicated in frames.
